# Relationship Between the Effect of Roxadustat and Comorbid Diabetes in Non-dialyzed Chronic Kidney Disease Patients: A Retrospective Observational Study

**DOI:** 10.7759/cureus.39543

**Published:** 2023-05-26

**Authors:** Hiroyuki Ito, Rie Araki, Toshiko Mori, Hideyuki Inoue, Suzuko Matsumoto, Shinichi Antoku, Tomoko Yamasaki, Michiko Togane

**Affiliations:** 1 Diabetes and Endocrinology, Edogawa Hospital, Tokyo, JPN; 2 Nephrology, Edogawa Hospital, Tokyo, JPN

**Keywords:** chronic kidney disease, renal anemia, diabetes, hypoxia-inducible factor prolyl hydroxylase inhibitor, roxadustat

## Abstract

Introduction

The dose of roxadustat, a hypoxia-inducible factor prolyl hydroxylase (HIF-PH) inhibitor, required to treat anemia, the hemoglobin level and the rate of hemoglobin target achievement were retrospectively investigated in non-dialyzed chronic kidney disease (CKD) patients with and without type 2 diabetes.

Methods

As the full analysis set, 25 subjects (10 with diabetes and 15 without diabetes) were observed over six months among 44 non-dialyzed CKD patients who received roxadustat. The target hemoglobin level was set at 110-130 g/L.

Results

The comorbidities of diabetes and body weight at baseline were significantly associated with each dose of roxadustat at six months and the change in each dose of roxadustat from the initiation of roxadustat treatment. There was no significant difference in the amount of increase in the hemoglobin level (14±11 g/L vs. 15±8 g/L) and the rate of hemoglobin target achievement (70% vs. 67%) between patients with and without diabetes. Each dose of roxadustat gradually decreased in patients without diabetes, whereas it increased in those with diabetes. Each dose of roxadustat was significantly higher in patients with diabetes than in those without diabetes at 3 (60±21 mg vs. 42±14 mg) and 6 (61±22 mg vs. 41±14 mg) months after the initiation of roxadustat treatment.

Conclusion

Roxadustat is useful for the treatment of anemia in both CKD patients with and without diabetes. However, the dose required to achieve the target hemoglobin level may be higher in patients with diabetes than in those without diabetes.

## Introduction

It is well known that anemia is closely associated with chronic kidney disease (CKD), cardiovascular diseases, and heart failure [1-3]. Due to the interaction of these pathological conditions, the concept of cardio-renal anemia (CRA) syndrome has been proposed [4]. Although it has been reported that the correction of anemia using an iron supplement and/or erythropoiesis-stimulating agent (ESA) improved not only the disturbed quality of life (QOL) but also the left ventricular hypertrophy [5,6] and the renal prognosis [7-10] in patients with CKD, the rate of hemoglobin target achievement remains inadequate in Japanese patients [11].

Cardiovascular diseases and end-stage kidney disease (ESKD) are major complications in patients with diabetes. Anemia is reportedly found more frequently in patients with diabetes than in the general population or nondiabetic CKD patients [12-17]. We also reported that even mild anemia, for which treatment was not indicated (hemoglobin level ≥110 g/L), was independently associated with diabetic microangiopathies and macroangiopathies based on a cross-sectional study of 1189 patients with type 2 diabetes [18]. Therefore, anemia is considered a serious problem in clinical practice related to managing type 2 diabetes, in which prevention of CKD progression and cardiovascular disease is important.

Hypoxia-inducible factor prolyl hydroxylase (HIF-PH) inhibitors have been available in Japan since 2019. Because roxadustat, a HIF-PH inhibitor, was covered by health insurance for renal anemia in non-dialyzed CKD patients in 2020, it is expected to improve the long-term prognosis of anemic patients due to its convenience as an oral drug. However, the effect of roxadustat may differ depending on patient background factors, and reports based on actual clinical practice are insufficient. Thus, we retrospectively investigated the factors that influence the effect of roxadustat in non-dialyzed CKD patients with renal anemia, with particular attention given to comorbid diabetes.

The primary outcome in the present study was the dose of roxadustat required to treat anemia in non-dialyzed CKD patients (hemoglobin level to start treatment: <110 g/L, target hemoglobin level: 110-130 g/L [19]) with and without type 2 diabetes. The secondary outcomes were the changes in hemoglobin, the rate of hemoglobin target achievement, and adverse events (AEs) during the six months of observation after the initiation of roxadustat.

## Materials and methods

**Figure 1:**
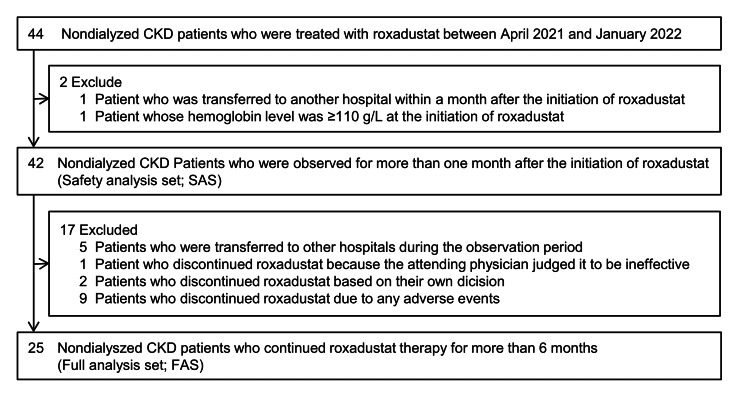
Flowchart of patient selection. The safety of roxadustat was analyzed in the safety analysis set (n=42), and the effectiveness was investigated in the full analysis set (n=25). CKD, chronic kidney disease

A flow chart of the patient selection process is shown in Figure 1. Forty-four Japanese non-dialyzed CKD patients with anemia who received roxadustat (Evrenzo® tablets; Astellas Pharma Inc., Tokyo, Japan) at our department from April 2021 to January 2022 were eligible for inclusion in this study. Subjects who were transferred to another hospital within a month after the initiation of roxadustat (n=1) and whose hemoglobin level was ≥110 g/L (n=1) at the time of the initiation of roxadustat were excluded from the analysis. In total, 42 patients with anemia were studied as the safety analysis set (SAS) to analyze the safety of roxadustat. After excluding subjects who were transferred to other hospitals during the observation period (n=5), those who discontinued roxadustat because the attending physician judged it to be ineffective (n=1), those who discontinued roxadustat based on the patient’s self-judgment (n=2), and those who discontinued roxadustat due to any AEs (n=9), 25 subjects were investigated as the full analysis set (FAS) to assess the effectiveness of roxadustat.

Roxadustat was administered orally thrice weekly at a two-day or three-day interval. When coadministered with phosphate binders or other medical products containing multivalent cations (e.g., calcium, iron, magnesium, or aluminum), patients were instructed to take roxadustat at least 1 hour before or after these concomitant medications. In principle, the initial dose of roxadustat was set to 50 mg for ESA-naïve patients and 70-100 mg for patients switching from ESAs. The initial dose of roxadustat was determined based on the judgment of the attending physician, who ensured that it did not exceed these doses.

The hemoglobin level and each dose of roxadustat during the 6 months after initiation were investigated in the FAS. The target hemoglobin level was set in the range of 110-130 g/L according to the guidelines for renal anemia issued by the Japanese Society for Dialysis Therapy [19]. Overshooting of the hemoglobin target was defined as a hemoglobin level of >130 g/L after the initiation of roxadustat.

The attending physician diagnosed the primary cause of CKD based on the patient’s medical history and/or histopathological findings from renal biopsy specimens. Diabetes was defined as a previous diagnosis of diabetes or using antidiabetic agents. The eGFR was calculated using the formula recommended by the Japanese Society of Nephrology [20].

The clinical parameters and AEs were retrospectively examined over six months after the initiation of roxadustat based on the subjects’ medical records.

Statistical analyses

All data are presented as the mean ± standard deviation. The χ2 test was used for between-group comparisons of categorical variables. Wilcoxon’s rank-sum test was used to assess the significance of differences in the hemoglobin level and each dose of roxadustat during the observation period compared to baseline values. The McNemar test was used to examine the changes over time in the rate of hemoglobin target achievement. A least squares model was used to evaluate the associations between the clinical background factors of the patients and each dose of roxadustat at 6 months, the change in each dose of roxadustat from the initiation, and the change in hemoglobin level. Factors that showed a significant association with each dependent variable in univariate analysis were included in a multivariate analysis. Odds ratios (ORs) and respective 95% confidence intervals (CIs) were determined by logistic regression analysis in order to examine the strength of the relationship between the clinical characteristics of patients and the achievement of target hemoglobin levels. P values of <0.05 (two-tailed) were considered to indicate statistical significance. The statistical software package JMP version 12.2.0 (SAS Institute, Cary, NC, USA) was used to perform all analyses.

## Results

The clinical characteristics of the FAS at baseline are shown in Table 1. Most of the subjects were over 65 years old. Diabetic nephropathy was diagnosed as the primary cause of CKD in 28%, and 40% (n=10) of the subjects had type 2 diabetes. Twenty-four percent of the FAS were taking iron supplements. The serum ferritin and transferrin saturation (TSAT) levels suggested that the iron storage was sufficient to initiate roxadustat. Statins or multivalent cations were used in 60% (n=6) and 73% (n=11) of patients with and without diabetes, respectively.

**Table 1:** The clinical characteristics of the full analysis set (n=25) at baseline ESA, erythropoiesis-stimulating agent; TSAT, transferrin saturation; eGFR, estimated glomerular filtration rate †Multivalent cations include preparations containing calcium, iron, magnesium or aluminum.

Clinical characteristics	Mean±SD/%
Age (years)	79±8
Age group (%)	
≤65 years	8
65-74 years	16
75-84 years	52
≥85 years	24
Male (%)	60
Primary cause of CKD (%)	
Diabetic nephropathy	28
Chronic glomerulonephritis	28
Nephrosclerosis	24
Others or unknown	20
Comorbid type 2 diabetes (%)	40
Switching from ESAs (%)	56
Concomitant medication (%)	
Iron	24
Multivalent cations†	32
Statins	52
Body weight (kg)	55.1±14.0
Systolic blood pressure (mmHg)	139±16
Diastolic blood pressure (mmHg)	66±12
Hemoglobin (g/L)	99±10
Serum iron (μmol/L)	15±5
TSAT (%)	32±11
Serum ferritin (μg/L)	211±144
Serum creatinine (μmol/L)	250±73
eGFR (mL/min/1.73 m^2^)	18±7
GFR stage (%)	
G3b/G4/G5	8/56/36
Starting dose of roxadustat (mg/dose)	53±11

**Figure 2:**
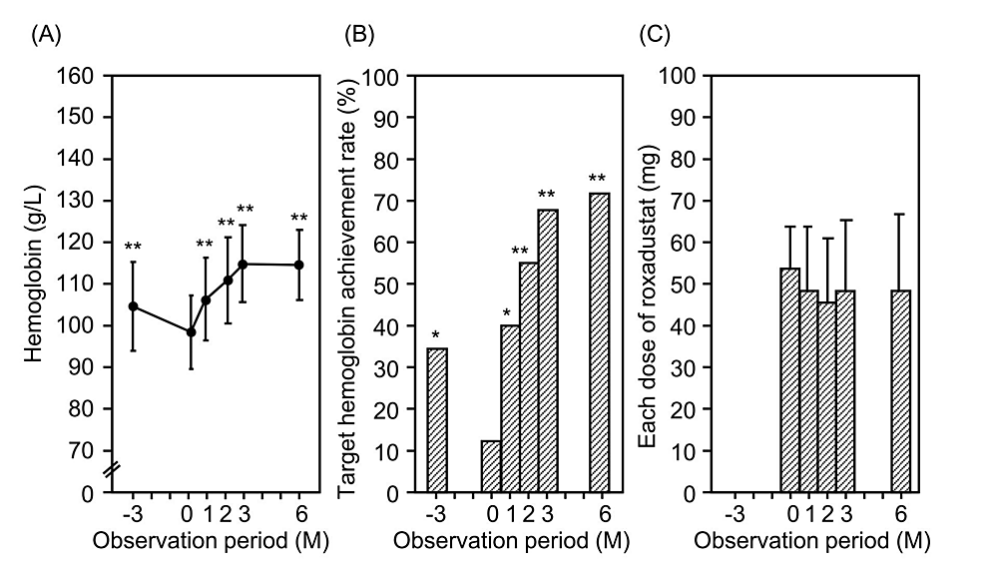
Changes in (A) the hemoglobin level, (B) the rate of hemoglobin target achievement, and (C) the each dose of roxadustat in the full analysis set. *P <0.05, **P <0.01 vs. baseline (0 month) value.

Figure 2 shows the changes in the hemoglobin level and rate of hemoglobin target achievement before and after the initiation of treatment and each dose of roxadustat. After the initiation of roxadustat, the hemoglobin level increased significantly from 99±10 g/L at the start of roxadustat to 114±8 g/L at 6 months. The amount of change in the hemoglobin level was 15±9 g/L. The rate of hemoglobin target achievement was 12% at baseline and 72% at six months after the initiation of roxadustat. Each dose of roxadustat showed a decrease from 53±11 mg at initiation to 49±20 mg at six months; however, this difference was not statistically significant. The amount of change in each dose of roxadustat was -4±23 mg.

The associations between the change in the hemoglobin level and the clinical parameters at baseline are shown in Table 2. The factors that showed a significant association with the change in hemoglobin level were switching from ESAs, concomitant use of iron, hemoglobin level, and serum ferritin level. In a multivariate analysis, the hemoglobin and serum ferritin levels tended to be associated with the change in the hemoglobin level.

**Table 2:** Associations between the change in hemoglobin level and the clinical parameters at baseline P, P-value; ESA, erythropoiesis-stimulating agent; TSAT, transferrin saturation; eGFR, estimated glomerular filtration rate †Multivalent cations include preparations containing calcium, iron, magnesium or aluminum.

Clinical parameters at baseline	Single regression		Multivariate regression	
	β	P	β	P
Age (/year)	-0.095	0.68		
Male sex	-0.267	0.94		
Diabetic nephropathy	-3.965	0.35		
Comorbidity of type 2 diabetes	-1.733	0.65		
Switching from ESAs	-8.435	0.02	10.008	0.30
Concomitant medication				
Iron	8.588	0.04	3.900	0.41
Multivalent cations†	6.412	0.10		
Statins	4.756	0.20		
Body weight (/kg)	-0.057	0.74		
Systolic blood pressure (/mmHg)	-0.129	0.40		
Diastolic blood pressure (/mmHg)	-0.209	0.29		
Hemoglobin (/g/L)	-0.616	<0.01	-0.539	0.07
Serum iron (/μmol/L)	0.946	0.15		
TSAT (/%)	0.590	0.03	-0.058	0.85
Serum ferritin (/μg/L)	0.046	<0.01	0.046	0.09
Serum creatinine (/μmol/L)	-0.112	0.66		
eGFR (/mL/min/1.73 m^2^)	-0.024	0.41		
Starting dose of roxadustat (/mg/dose)	-0.238	0.18		

The associations between the rate of hemoglobin target achievement at six months after the initiation of roxadustat and the clinical parameters at baseline are shown in Table 3. None of the baseline characteristics of the subjects were significantly associated with the rate of hemoglobin target achievement.

**Table 3:** Associations between the rate of hemoglobin target achievement at 6 months after the initiation of roxadustat and the clinical parameters at baseline OR, Odds ratio; CI, confidence interval; P, P-value; ESA, erythropoiesis-stimulating agent; TSAT, transferrin saturation; eGFR, estimated glomerular filtration rate †Multivalent cations include preparations containing calcium, iron, magnesium, or aluminum.

Clinical parameters at baseline	Single regression	
	OR [95% CI]	P
Age (/year)	1.06 [0.96-1.19]	0.24
Male sex	0.94 [0.15-5.19]	0.95
Diabetic nephropathy	0.64 [0.11-4.06]	0.62
Comorbidity of type 2 diabetes	0.64 [0.11-3.50]	0.60
Switching from ESAs	0.33 [0.04-1.91]	0.22
Concomitant medication		
Iron	2.69 [0.34-57.03]	0.37
Multivalent cations†	1.50 [0.25-12.40]	0.67
Statins	4.71 [0.82-38.99]	0.08
Body weight (/kg)	0.94 [0.85-1.02]	0.15
Systolic blood pressure (/mmHg)	0.99 [0.92-1.05]	0.65
Diastolic blood pressure (/mmHg)	0.95 [0.85-1.04]	0.26
Hemoglobin (/g/L)	1.08 [0.99-1.21]	0.08
Serum iron (/μmol/L)	0.97 [0.79-1.21]	0.76
TSAT (/%)	0.99 [0.89-1.10]	0.80
Serum ferritin (/μg/L)	1.00 [0.99-1.01]	0.97
Serum creatinine (/μmol/L)	1.00 [0.99-1.01]	0.72
eGFR (/mL/min/1.73 m^2^)	0.96 [0.84-1.04]	0.51
Starting dose of roxadustat (/mg/dose)	1.56 [incalculable]	0.21

The associations between each dose of roxadustat at six months after initiation and the clinical parameters at baseline are shown in Table 4. Diabetic nephropathy as the primary cause of CKD, comorbid type 2 diabetes, and body weight were significantly associated with each dose of roxadustat at six months in univariate analyses. Because diabetic nephropathy and comorbid type 2 diabetes are obviously confounding factors, a multivariate analysis was performed using the comorbid type 2 diabetes and body weight as independent variables. As a result, body weight was a significant explanatory factor for each dose of roxadustat at six months, and comorbid type 2 diabetes tended to be associated with this dose.

**Table 4:** Associations between each dose of roxadustat at 6 months after the initiation of roxadustat and the clinical parameters at baseline P, P-value; ESA, erythropoiesis-stimulating agent; TSAT, transferrin saturation; eGFR, estimated glomerular filtration rate †Multivalent cations include preparations containing calcium, iron, magnesium, or aluminum.

Clinical parameters at baseline	Single regression		Multivariate regression	
	β	P	β	P
Age (/year)	-0.088	0.86		
Male sex	7.949	0.14		
Diabetic nephropathy	18.968	0.03		
Comorbidity of type 2 diabetes	19.667	0.01	19.359	0.06
Switching from ESAs	11.558	0.16		
Concomitant medication				
Iron	-1.140	0.91		
Multivalent cations†	-0.662	0.94		
Statins	-6.346	0.44		
Body weight (/kg)	1.050	<0.01	0.819	0.03
Systolic blood pressure (/mmHg)	0.508	0.18		
Diastolic blood pressure (/mmHg)	0.496	0.32		
Hemoglobin (/g/L)	-0.094	0.83		
Serum iron (/μmol/L)	-0.240	0.83		
TSAT (/%)	-0.122	0.81		
Serum ferritin (/μg/L)	-0.028	0.51		
Serum creatinine (/μmol/L)	0.006	0.92		
eGFR (/mL/min/1.73 m^2^)	0.959	0.12		
Starting dose of roxadustat (/mg/dose)	-0.164	0.68		

The associations between the change in each dose of roxadustat at six months from initiation and the clinical parameters at baseline are shown in Table 5. Comorbid type 2 diabetes and body weight were significantly associated with changes in each dose of roxadustat in both univariate and multivariate analyses.

**Table 5:** Associations between the change in each dose of roxadustat 6 months after the initiation of roxadustat and the clinical parameters at baseline P, P-value; ESA, erythropoiesis-stimulating agent; TSAT, transferrin saturation; eGFR, estimated glomerular filtration rate †Multivalent cations include preparations containing calcium, iron, magnesium, or aluminum.

Clinical parameters at baseline	Single regression		Multivariate regression	
	β	P	β	P
Age (/year)	-0.357	0.54		
Male sex	10.667	0.27		
Diabetic nephropathy	18.889	0.07		
Comorbidity of type 2 diabetes	21.000	0.03	22.05	0.049
Switching from ESAs	6.558	0.50		
Concomitant medication				
Iron	-0.022	0.92		
Multivalent cations†	-0.221	0.98		
Statins	-3.718	0.70		
Body weight (/kg)	1.029	0.01	0.802	0.04
Systolic blood pressure (/mmHg)	0.458	0.24		
Diastolic blood pressure (/mmHg)	0.455	0.38		
Hemoglobin (/g/L)	-0.372	0.46		
Serum iron (/μmol/L)	-0.011	0.99		
TSAT (/%)	0.113	0.83		
Serum ferritin (/μg/L)	-0.028	0.51		
Serum creatinine (/μmol/L)	-0.051	0.44		
eGFR (/mL/min/1.73 m^2^)	1.205	0.10		
Starting dose of roxadustat (/mg/dose)	-1.164	<0.01	-1.736	0.095

**Figure 3:**
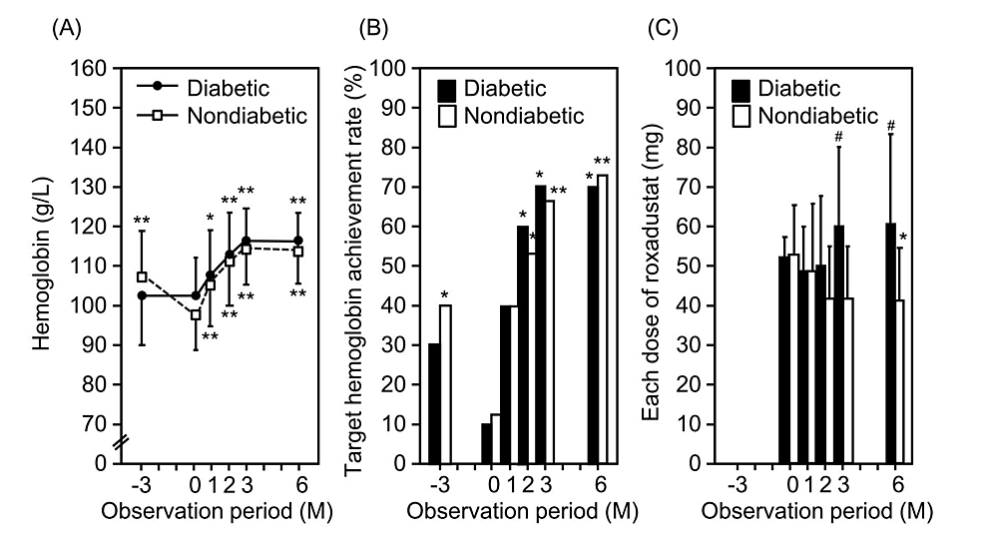
Changes in (A) the hemoglobin level, (B) the rate of hemoglobin target achievement, and (C) the each dose of roxadustat in subjects with and without diabetes in the full analysis set. Closed circles and bars indicate subjects with diabetes. Open squares and bars indicate those without diabetes. *P <0.05, **P <0.01 vs. corresponding value at baseline (0 month) value. #P <0.05 vs. subjects without diabetes.

The baseline clinical characteristics of patients with and without diabetes are shown in Table 6. No characteristics differed between the two groups. Figure 3 and Table 7 show the changes in hemoglobin level and the rate of hemoglobin target achievement before and after the initiation of roxadustat and each dose of roxadustat after the initiation of roxadustat in patients with (n=10) and without (n=15) type 2 diabetes. There was no difference between the two groups in the amount of change in the hemoglobin level (14±11 g/L vs. 15±8 g/L) or the rate of hemoglobin target achievement (70% vs. 67% at six months after the initiation of roxadustat). However, each dose of roxadustat tended to decrease gradually in patients without diabetes, whereas it increased in those with diabetes. As a result, each dose of roxadustat was significantly higher in patients with diabetes than in patients without diabetes at three (60±21 mg vs. 42±14 mg, P=0.02) and six months (61±22 mg vs. 41±14 mg, p=0.01) after the initiation of roxadustat. The amount change in each dose of roxadustat was also significantly higher in patients with diabetes than in patients without diabetes (9±24 mg vs. -12±19 mg, p=0.03).

**Table 6:** Clinical characteristics in patients with and without diabetes at baseline P, P-value; ESA, erythropoiesis-stimulating agent; TSAT, transferrin saturation; eGFR, estimated glomerular filtration rate †Multivalent cations include preparations containing calcium, iron, magnesium, or aluminum.

Clinical characteristics	Diabetes	No diabetes	P
n	10	15	
Age (years)	76±8	80±8	0.12
Male (%)	80	47	0.10
Switching from ESAs (%)	70	47	0.25
Concomitant medication (%)			
Iron	20	27	0.70
Multivalent cations†	30	33	0.86
Statins	60	47	0.51
Body weight (kg)	61±16	52±12	0.23
Systolic blood pressure (mmHg)	142±8	136±20	0.19
Diastolic blood pressure (mmHg)	64±16	69±10	0.51
Serum iron (μmol/L)	18±4	14±4	0.13
TSAT (%)	35±13	30±11	0.45
Serum ferritin (μg/L)	205±246	212±135	0.81
Serum creatinine (μmol/L)	249±77	251±73	0.82
eGFR (mL/min/1.73 m^2^)	20±8	17±5	0.34
GFR stage (%)			
G3b/G4/G5	20/50/30	0/60/40	0.19

**Table 7:** Comparison of the changes in the hemoglobin levels, the rate of hemoglobin target achievement, and the each doses of roxadustat in patients with and without diabetes P, P-value *P <0.05, **P <0.01 vs. corresponding value at baseline

Period	Hemoglobin (g/L)			Rate of hemoglobin target achievement (%)			Each dose of roxadustat (mg)		
	Diabetes	No diabetes	P	Diabetes	No diabetes	P	Diabetes	No diabetes	P
-3 months	102±12	107±12**	0.28	30	40*	0.61			
Baseline	102±8	98±10	0.39	10	13	0.80	52±6	53±13	0.81
1 month	109±11*	107±11**	0.54	40	40	1.00	49±12	49±18	0.75
2 months	113±11**	111±12**	0.60	60*	53*	0.74	53±15	42±14	0.05
3 months	116±9**	114±10**	0.56	70*	67*	0.86	60±21	42±14	0.02
6 months	115±7**	113±8**	0.36	70*	73*	0.86	61±22	41±14	0.01
Amount of change	14±11	15±8	0.34				9±24	-12±19	0.03

In the SAS (male, 60%; age, 80±9 years; hemoglobin level at the initiation of roxadustat, 98±10 g/L, each dose of roxadustat, 58±17 mg), AEs were observed in 30 patients (71%), for a total of 40 events. The AEs observed in >5% of the subjects included hyperkalemia (n=5), progression of renal dysfunction (n=3), overshooting of hemoglobin (n=3), edema (n=3), nausea (n=3), constipation (n=3) and dementia (n=3). The clinical characteristics of 9 subjects who discontinued roxadustat due to AEs are shown in Table 8. Most AEs leading to discontinuation of roxadustat occurred within a short period after the initiation of roxadustat and were not associated with patient sex, each dose of roxadustat or comorbid diabetes (Table 9).

**Table 8:** All adverse events in the safety analysis set †Hemoglobin level >130g/L

Adverse event	N (%)
Patients with at least one adverse event	30 (71)
Hyperkalemia	5 (12)
Progression of renal impairment	3 (7)
Overshooting of hemoglobin target†	3 (7)
Edema	3 (7)
Nausea	3 (7)
Constipation	3 (7)
Dementia	3 (7)
Anorexia	2 (5)
Heart failure	2 (5)
Pneumonia (death)	1 (2)
Lung cancer	1 (2)
Sprain	1 (2)
Deep vein thrombosis	1 (2)
Cataract	1 (2)
Gait disturbance	1 (2)
Chest pain	1 (2)
Headache	1 (2)
Elevation of blood pressure	1 (2)
Decline of blood pressure	1 (2)
Choledocholithiasis	1 (2)
Dizziness	1 (2)
Stiff shoulder	1 (2)

**Table 9:** Clinical characteristics of the subjects who discontinued roxadustat according to the adverse events Hb, hemoglobin †Period from the roxadustat initiation ‡ Each dose of roxadustat at the discontinuation

Period† (months)	Age (years)	Sex	Dose‡(mg)	Diabetes	Cause of discontinuation
<1	80	Female	100	Absent	Nausea
<1	83	Female	100	Absent	Deep vein thrombosis
<1	85	Male	50	Present	Overshooting of Hb (143 g/L)
<1	62	Male	50	Present	Overshooting of Hb (145 g/L)
<1	88	Female	50	Absent	Stiff shoulder
<1	100	Male	50	Absent	Edema
1-2	75	Female	50	Present	Edema
1-2	80	Male	70	Present	Overshooting of Hb (152 g/L)
2-3	76	Male	50	Present	Pneumonia (death)

## Discussion

In the present study, roxadustat therapy was effective for anemia in non-dialyzed CKD patients in the short term, regardless of patient age, a primary cause of CKD, presence of comorbid diabetes, body weight, eGFR and starting dose of roxadustat. According to single regression analyses, the factors associated with the change in hemoglobin after the administration of roxadustat included switching from ESAs, concomitant use of iron, hemoglobin level, and serum ferritin level. These findings are considered important for predicting the therapeutic response when starting HIF-PH inhibitors and may be useful in avoiding the risk of overshooting hemoglobin and thrombosis. Consideration, such as reducing the starting dose of roxadustat, may be necessary, especially for patients who have not used an ESA and/or who are on iron supplementation.

Each dose of roxadustat at six months was significantly higher in patients with type 2 diabetes than in those without diabetes in the present study. CKD patients with diabetes are known to have multiple causes of anemia, resulting in a higher incidence of anemia in comparison to nondiabetic subjects [14-16]. Decreased EPO production is one of the reasons for the high frequency of anemia in diabetic patients. HIF-PH inhibitors increase endogenous EPO production by stabilizing the HIF-α subunit and enhancing the hypoxia-associated EPO gene expression by dimerizing with the HIF-ß subunit. HIF-PH inhibitors are also known to improve iron utilization through the inhibition of hepcidin production [21]. Akizawa et al. [22] reported, based on a phase 3 study, that roxadustat maintained hemoglobin levels in the target range independently of comorbid diabetes in non-dialyzed CKD patients with anemia.

Furthermore, Zhang et al. [23] reported, based on a retrospective cohort study with a three-month observation period, that the effect of roxadustat on improving renal anemia was similar to that of ESAs in 61 patients with diabetic kidney disease. Hirai et al. [24] also retrospectively demonstrated that switching from ESAs to roxadustat further improved hemoglobin regardless of the presence of diabetes and was accompanied by an improvement in lipid metabolism in 50 non-dialyzed CKD patients. These reports suggested that roxadustat therapy can improve renal anemia in patients with diabetes and nondiabetic subjects, similar to the results obtained from the present study. However, the dose of roxadustat required to achieve target hemoglobin levels in patients with diabetes has not been clarified in actual practice. Therefore, the present study is considered useful for predicting the change in hemoglobin and the dose of roxadustat required in CKD patients with type 2 diabetes and anemia, although this study included a small number of subjects.

According to a phase 3 trial, the dose of roxadustat was larger in non-dialyzed CKD patients with diabetes (53.75±23.50 mg) than in those without diabetes (49.71±20.67 mg); however, the difference was not statistically significant (P=0.299) [25]. The target hemoglobin level was the greatest difference between the present study and the phase 3 trial [23,26]. While roxadustat was administered to maintain hemoglobin levels within 110-130 g/L in the present study, according to the current guidelines for the treatment of renal anemia in non-dialyzed CKD patients [19], the target hemoglobin level was set between 100-120 g/L in the phase 3 trial. In addition, the baseline hemoglobin levels differed between the present study (102±8 g/L in debates patients and 98±10 g/L in nondiabetic patients) and the phase 3 trial (110.5±5.8 g/L in diabetes patients and 110.2±5.4 g/L in nondiabetic patients) because the study subjects were treated by switching from ESAs. These differences may have contributed to the different results between the two studies. In the present study, the factors that affected the dose of roxadustat were body weight and comorbid type 2 diabetes. Although not significant, the body weight was greater in patients with diabetes than in those without diabetes. This 9 kg weight difference may have also been associated with the higher dose of roxadustat in patients with diabetes than those without diabetes in this study. As patients with diabetes are commonly heavier than nondiabetic patients, it may be necessary to take body weight into consideration when adjusting the subsequent dose of roxadustat after the initiation of treatment.

Limitations of the study

The present study was associated with several limitations. First, it was a retrospective observational investigation of a small number of patients. Thus, it is necessary to note the possibility that the changes in hemoglobin and the dose of roxadustat occurred incidentally due to the low statistical power. Additionally, the statistical analysis of the continuous outcomes may not be appropriate if the data does not meet the assumptions of the least squares model. Further investigations on a larger number of patients should be performed to confirm our results. The target hemoglobin level differs depending on the guidelines. For example, KDIGO recommendations consist of a target hemoglobin level of 100-115 g/L in patients undergoing ESA treatment [27]. According to the European Renal Best Practice (ERBP), the treatment should target 100-110 g/L ranges of hemoglobin [28]. It is necessary to recognize this variation in the target hemoglobin level may affect the result of drug treatment for anemia in CKD patients. Second, the present study could not determine medication adherence to pharmacological therapy. Due to its half-life, roxadustat is prescribed to be administered three times weekly to both dialyzed and non-dialyzed CKD patients [26]. Concomitant use with statins or multivalent cations may inhibit absorption due to chelate formation, resulting in a decrease in the blood concentration of roxadustat [29]. There was no significant difference in the frequency of concomitant use of statins or multivalent cations between patients with and without diabetes. Therefore, it is necessary to pay attention to the timing of roxadustat administration in CKD patients who often develop polypharmacy. In this study, we instructed the subjects on the timing of taking roxadustat; however, there is a concern that medication adherence may have been insufficient. If this medication adherence was poor in the subjects with diabetes, as previously reported [30], it is not surprising that patients with diabetes required a higher dose of roxadustat to maintain their target hemoglobin level. The fact that this adherence was not evaluated is considered to be another limitation of this study.

Despite these limitations, we believe that roxadustat is effective for treating anemia in non-dialyzed CKD patients with diabetes, and administration in higher doses may be needed compared to patients without diabetes. The findings obtained from this study are considered valuable as real-world data.

## Conclusions

Roxadustat is useful for the treatment of anemia in non-dialyzed CKD patients. However, the doses required to maintain the target hemoglobin levels may differ between patients with and without diabetes.
